# Application of FA-CGP-GGBS Geopolymers as Cement Substitute in Crushed Stone Base

**DOI:** 10.3390/ma19050829

**Published:** 2026-02-24

**Authors:** Xiaozhe Wang, Ling Luo, Yongjun Qin, Zhenji Long

**Affiliations:** 1 School of Civil Engineering and Architecture, Xinjiang University, Urumqi 830047, China; 2 Xinjiang Civil Engineering Technology Research Center, Xinjiang University, Urumqi 830047, China

**Keywords:** geopolymers, recycled aggregate, mechanical properties, durability, microstructure

## Abstract

In this study, a geopolymer was prepared using a sodium silicate solution to enhance the activity of coal gangue powder (CGP), fly ash (FA), and granulated blast furnace slag powder (GGBS) for replacing cement in the crushed stone base. The unconfined compressive strength, splitting tensile strength, water stability, and erosion resistance of geopolymers containing precursor contents (5%, 6%, 7%) were analyzed, and the reaction mechanism was systematically studied in combination with MIP, SEM, XRD, and TG. The cement content was 7%. The results indicate that the unconfined compressive strength and splitting tensile strength of the geopolymer mixture increased by 7.58% and 9.52%, respectively, compared with those of the cement mixture. The strength loss rate due to water stability and erosion resistance is reduced by 1.000% and 0.315%, respectively, compared with the cementitious system at the exact 7% cementitious material dosage. Compared with the cementitious system, the geopolymer system shows an 8% increase in harmless pores, a 10% reduction in harmful pores, and a 4.29% reduction in mass loss rate. This study proposes a new approach to utilizing solid waste resources and provides a theoretical basis for its application in gravel-based systems.

## 1. Introduction

Industrial solid waste refers to various waste residues, dust, and other wastes discharged into the environment during the production process of various industrial enterprises. China, as a major industrial nation, generates vast quantities of fly ash (FA) and industrial waste slag annually, with an accumulated volume of approximately 3.68 billion tons per year and a low utilization rate [1]. Aluminosilicate polymers produced from various industrial solid waste materials are gradually emerging as a practical utilization pathway [2]. As an alternative to cementitious materials, geopolymers exhibit excellent durability, high mechanical strength, and low shrinkage, making them well-suited for broad engineering applications [3,4].

Many scholars have studied geopolymers of fly ash-granulated blast furnace slag. Zhang et al. [5] showed that alkali-activated concrete had higher mechanical strength, greater chloride diffusion resistance, and lower porosity than ordinary concrete. Wei et al. [6] prepared a new type of recycled concrete using steel slag, fly ash, and mineral powder. They compared it with traditional concrete and found that when the fly ash content was about 30%, higher unconfined compressive strength could be obtained. Sasui S et al. [7] investigated the effects of different Na_2_SiO_3_:NaOH ratios and different NaOH molar concentrations on the strength and chloride-binding capacity of fly ash/granulated blast furnace slag concrete. They concluded that the optimal catalyst solution formulation achieves a molar ratio of Na_2_O to SiO_2_ of 1, with an acceptable error range of ±3%. Emarah A D et al. [8] used fly ash and blast furnace slag powder as cementitious substances in aerated concrete, and used four prediction models to predict the compressive strength. The results showed that the artificial neural network performed optimally. The influencing factors were curing temperature > ratio of alkaline solution to binder (AL/b) > NaOH concentration > and sample age. Feng et al. [9] partially replaced granulated blast furnace slag powder with phosphogypsum to prepare geopolymers and found that phosphogypsum could promote the alkali activation reaction and delay the formation of hydration products, but reduced the working performance of the alkali activation system. Liu et al. [10] used calcium carbide slag as an activator to modify fly ash/granulated blast furnace slag geopolymers, and determined that the GGBS-to-FA ratio of 8:2 is the best mass ratio, the optimal amount of calcium carbide slag is 8%, and the strength is the highest.

As a coal-based solid waste, China’s storage capacity for coal gangue is about 6–7 billion tons, and it continues to grow at an astonishing rate of more than 300 million tons per year [11]. At present, there is no effective treatment method for coal gangue, and its overall utilization rate is low. Still, the researchers found that the coal gangue powder (CGP) powder obtained by high-temperature calcination combined with mechanical grinding has high volcanic ash activity and can be used to prepare geopolymer cementitious materials [12,13]. Wu et al. [14] subjected coal gangue powder to pre-wetting treatment and prepared cementitious mortars with cement and fly ash. They analyzed the effects of coal gangue powder on the hydration process, shrinkage behavior, and mechanical properties of the mortars. Pre-wetted coal gangue powder reduced mortar shrinkage and enhanced compressive strength. Song et al. [15] used coal gangue powder, circulating fluidized bed fly ash, and carbide slag to prepare grouting materials. When the coal gangue powder content was 20%, the slurry exhibited excellent thermal stability and could be used in grouting engineering.

In current road construction, ordinary Portland cement (OPC) remains the primary binder [16], and its production and use account for 7% of global greenhouse gas emissions each year [17]. In the context of energy conservation and emission reduction, many scholars are seeking alternatives to cement products, and geopolymers, as a green and sustainable building material, have significant application potential [18]. Yuan et al. [19] used lithium slag as a cement substitute in crushed stone bases and found that it exhibits significant reactivity. It synergistically reacts with cement and, at appropriate dosages, enhances mechanical properties, durability, and shrinkage resistance. Bao et al. [20] used fly ash (FA) and slag powder (SP) to prepare geopolymers for application in semi-rigid base layers, and found that geopolymer stabilized gravel (GSG) exhibited excellent resistance to expansion and shrinkage, while reducing the carbon footprint. Li et al. [21] sought to prepare geopolymers that partially replaced cement. They jointly produced a geopolymer-cement-stabilized aggregate mixture and conducted in-depth research on the mineral powder, fly ash, and wet calcium carbide residue used as precursor materials, focusing on the variation in their strength under freeze-thaw cycles and the mechanisms underlying these variations. Tests showed that the mixture exhibited optimal freeze resistance at a geopolymer content of 20%. Gao et al. [22] modified fly ash geopolymers using steel slag as a binder for stabilizing gravel aggregates, evaluating their mechanical properties and water stability. The incorporation of steel slag not only enhanced the mixture’s strength and water stability but also increased the reactivity of the entire system. Hu et al. [23] investigated the effects of two key factors—temperature and moisture—on a geopolymer-stabilized aggregate base prepared from fly ash and red mud. They found that increasing the temperature from 20 °C to 38 °C positively influenced the strength development of the specimens, whereas reduced humidity negatively impacted specimen strength. This indicates that geopolymer-stabilized subbases still require moist conditions. Yue et al. [24] found that geopolymer-stabilized crushed stone exhibited superior dry shrinkage properties compared to cement-fly-ash-stabilized crushed stone. Microscopic testing revealed no significant porosity around the ITZ in the geopolymer matrix, with excellent bonding between the polymers and aggregates. The development of geopolymers as cementitious materials not only consumes large amounts of industrial solid waste but also improves the utilization of these resources, achieving energy conservation and emission-reduction goals and reducing the carbon footprint [25]. In summary, numerous scholars have investigated the fundamental properties of geopolymers as cement substitutes, yet research on their water resistance remains limited. This study, therefore, examines the mechanical properties and water resistance of multi-component geopolymer systems, providing technical support for the comprehensive application of solid waste-based green building materials in road base engineering.

In this study, FA, GGBS, and CGP are discussed for the preparation of a geopolymer concrete suitable for fully recycled crushed-stone base applications. Based on the recommended binder content (5–9%) specified in standard JTG/TF20-2015 [26], the binder contents selected for this study are 5%, 6%, and 7%. By testing the unconfined compressive strength and splitting tensile strength of geopolymers with varying binder dosages and comparing them against a 7% cement binder dosage, this study analyzes the applicability and feasibility of FA-GGBS-CGP geopolymers in fully recycled crushed stone base courses. Subsequently, water stability and erosion resistance are examined to demonstrate further the application potential of geopolymer systems over cement-based systems. Finally, the micro-reaction mechanisms between geopolymers were investigated using mercury intrusion porosimetry (MIP, Micromeritics Autopore V 9505, Micromeritics, Norcross, GA, USA), scanning electron microscopy (SEM, Hitachi SU8600, HITACHI, Tokyo, Japan), X-ray diffraction (XRD, Siemens D8, Bruker, Karlsruhe, Germany), and thermogravimetric-differential thermal analysis (TG-DTG, HITACHI STA7300, HITACHI, Tokyo, Japan).

## 2. Materials and Methods

### 2.1. Test Materials

#### 2.1.1. Cementitious Materials

**Figure 1:**
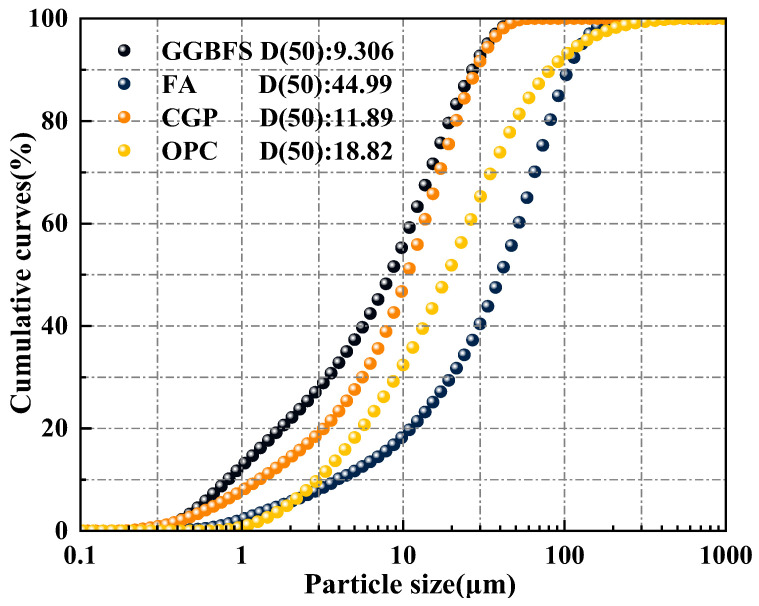
Particle size distribution of precursor materials.

Coal gangue powder (CGP) is produced by Hebei Kexu Building Materials Co., Ltd. (Shijiazhuang, China) through low-temperature calcination (650–750 °C) to achieve 800 mesh fineness. Grade II fly ash (FA) and ground-granulated blast-furnace slag (GGBS) are sourced from the Bayi Iron and Steel Plant in Urumqi, Xinjiang. P·O 42.5 ordinary Portland cement (OPC), branded as Tianshan, is manufactured in Changji, Xinjiang, China. Figure 1 shows the particle size analysis of the three precursor materials and cement, while Table 1 presents the average test results for X-ray fluorescence spectrometry (XRF, Rigaku ZSX Primus III+, Rigaku Corporation, Tokyo, Japan) and loss on ignition (LOI).

#### 2.1.2. Alkali Activator

The alkali-activated system is based on a liquid sodium silicate solution (Na_2_O·nSiO_2_) and uses caustic soda (NaOH) to adjust the modulus of the alkali activation solution. The activator modulus is 1.5 M. Sodium silicate is prepared from an industrial-grade water glass solution, with specific parameters as shown in Table 2. Uses flake NaOH (analytically pure) with a purity ≥ 96%.

#### 2.1.3. Recycled Aggregates

**Figure 2:**
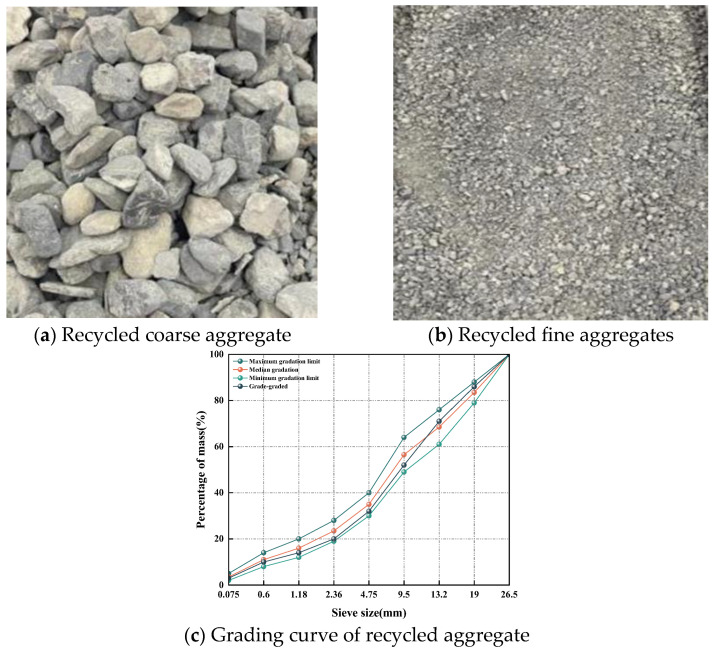
Recycled aggregate sample and gradation curve.

Recycled aggregate (RA) is obtained from the reconstruction and demolition of the abandoned road concrete after the reconstruction and demolition of the apron of a military garrison in Liu Gong Town, Changji Prefecture, Xinjiang, through crushers and other professional equipment. Recycled aggregate (RA) and the gradation curve are shown in Figure 2.

#### 2.1.4. Mix Design

Specimens were molded in accordance with JTG3441-2024 standard [27]. The specific quantities of each component are listed in Table 3.

### 2.2. Test Method

#### 2.2.1. Mechanical Properties

**Figure 3:**
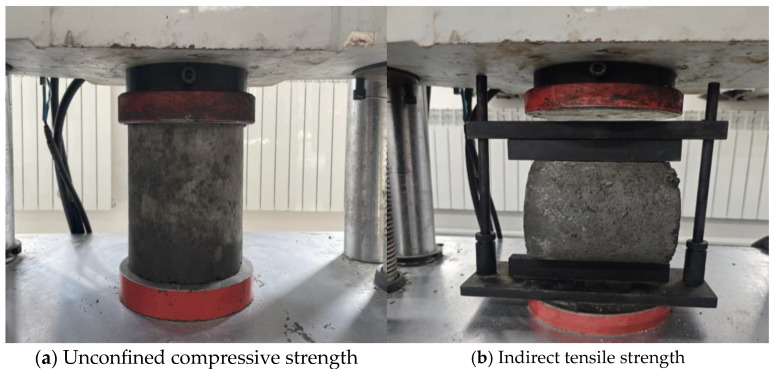
Samples test photos.

First, prepare 13 cylindrical standard specimens per group, each with dimensions of Ф150 mm × 150 mm. Cure the specimens under standard conditions (sealed, 20 ± 2 °C, ≥95% relative humidity). After curing, test the unconfined compressive strength (UCS) and indirect tensile strength (ITS) of the specimens in accordance with the ‘Test Regulations for Stabilizing Materials of Inorganic Binders in Highway Engineering’ JTG3441-2024. Use an electro-hydraulic servo universal testing machine, set the loading rate to 1 mm/min, and continue pressurizing until specimen failure. Sample testing photos are shown in Figure 3.

#### 2.2.2. Water Stability

Water stability is determined by measuring the UCS of specimens after prolonged immersion and comparing it with that of specimens cured under standard conditions at the same age. For each test group, six parallel specimens are tested. Moisture stability is calculated as the ratio of the immersed UCS to the standard-cured UCS, while strength loss is calculated as the percentage decrease from the standard-cured UCS. The calculation formulas are as shown in (1) and (2).

(1) $$K_{r}=\frac{R_{ct}}{R_{co}}$$

(2) $${∆}_{R}=\frac{R_{co}-R_{ct}}{R_{co}}\times 100\%$$

*K_r_*—Water stability coefficient;

*R_ct_*—Unconfined compressive strength of specimens after water immersion curing/MPa;

*R_co_*—Unconfined compressive strength of specimens after standard curing/MPa;

∆*_R_*—Strength loss/%.

#### 2.2.3. Scouring Resistance

The scouring resistance test was conducted in accordance with the specification “Test Regulations for Stabilized Materials of Inorganic Adhesives for Highway Engineering” (JTG3441-2024), and 6 beam specimens measuring 100 mm × 100 mm × 400 mm were prepared in each group. The test results were averaged across the six specimen groups.

### 2.3. Microscopic Testing

#### 2.3.1. MIP

Mortar samples terminated by anhydrous ethanol were quantitatively analyzed by mercury intrusion porosity (MIP) for airstrike structural parameters (such as porosity, pore size, and pore size distribution) in the matrix, and tested with the AutoPoreIV9510 instrument (Micromeritics, Norcross, GA, USA).

#### 2.3.2. SEM

After gold spraying, mortar samples were examined under a Hitachi SU8600 (HITACHI, Tokyo, Japan) to observe the microscopic morphology of different test groups.

#### 2.3.3. XRD

After the slurry samples to be tested were ground into powder, the microstructure and phase of the samples from different test groups were analyzed using a Siemens D8 advanced X-Ray diffractometer (Bruker, Karlsruhe, Germany) with Cu Kα radiation. The target was a copper target, the scanning speed was 5°/min, and the 2θ range was 5–80°. XRD results were analyzed using MDI JaDe6 (V6) software.

#### 2.3.4. TG-DTG

Slurry samples that have been terminated in hydration are ground into powder, dried, and tested using a HITACHI STA7300 thermal analyzer (HITACHI, Tokyo, Japan) in the range of 25 °C~900 °C. At a heating rate of 10 °C/min in a nitrogen atmosphere.

## 3. Results and Analysis

### 3.1. Mechanical Properties

#### 3.1.1. UCS

**Figure 4:**
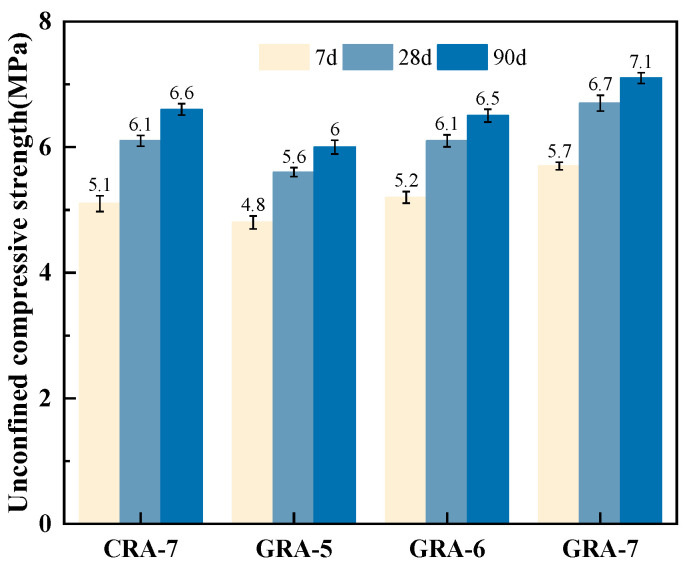
Unconfined compressive strength of the samples.

Figure 4 shows the UCS results for the 4 mixes. The experimental results showed that increasing the geopolymer cementitious material content increased the UCS of the sample. Taking the UCS at 90 days as an example, the UCS of the GRA-7 group reached a maximum of 7.1 MPa, which was 18.33% and 9.23% higher than that of the GRA-5 and GRA-6 groups under the same cementitious material condition, and 7.58% higher than that of the CRA-7 group under the exact dosage but different materials. With increased content of cementitious material and extended curing time, the geopolymerization reaction proceeds sufficiently, generating more gel phases, which in turn improve the degree of mutual cementation between the internal components and the later strength of the material [28]. Geopolymer precursor material dissolves in an alkaline environment. This process releases silicate and aluminate tetrahedra. These units then undergo polymerization, forming geopolymer gel products. Furthermore, the highly alkaline environment erodes the residual cement mortar on the recycled aggregate’s surface. The generated gel phase thereby creates a solid connection between the geopolymer binder and the recycled aggregate [29]. Simultaneously, the recycled aggregate has a rough and porous surface. Internal defects are also present due to the secondary crushing process. Consequently, multiple microcracks are present within the aggregate [30]. The hydration products from the geopolymer cementitious material can fill some of these surface defects [31]. This mechanism results in the geopolymer-stabilized recycled mixture developing higher strength than cement-stabilized recycled aggregate, even at the exact dosage.

When the local polymer content exceeds 5%, the 7-day unconfined compressive strength meets the heavy traffic standards for expressways and first-class highways (≥4.0 MPa). At a content of 6% or higher, it meets the extremely heavy and super-heavy traffic standards for expressways and first-class highways (≥5.0 MPa).

#### 3.1.2. ITS

**Figure 5:**
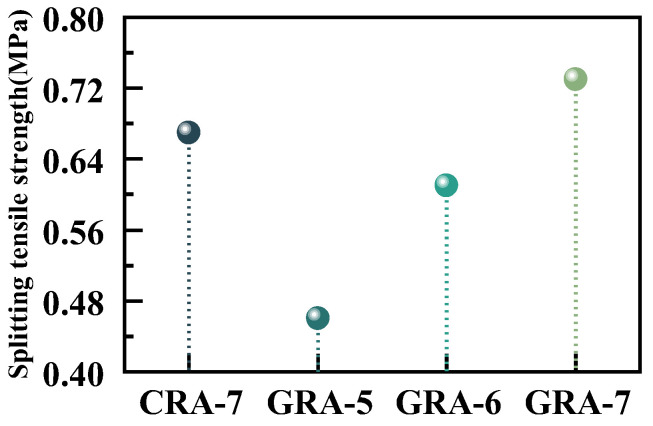
Splitting the tensile strength of the samples.

Figure 5 shows the ITS test results for the four mixture groups. ITS presents the same changes as UCS. With the increase in geopolymer cementitious material content from 5% to 7%, the ITS of the GRA-7 group increased by 0.27 MPa compared with the GRA-5 group, and the GRA-7 group’s strength was higher than that of the CRA-7 group at the duplicate 7% cementitious material content. The increase in the content of geopolymer materials leads to the formation of more hydration products, resulting in a stable connection between the cement material and the recycled aggregate, and the ITS of the geopolymer-stabilized recycled aggregate is significantly improved [32]. At the exact dosage, old mortar flakes off the surface of recycled aggregates. Some silicon and aluminum elements from this old mortar can also participate in the hydration reaction [33]. Compared with cement, the resulting gel products adhere more strongly to particle surfaces. As the geopolymer content increases and curing time extends, the thickness of the reaction product layer surrounding the recycled aggregate particles gradually increases, filling the pores and narrowing the interfacial transition zone (ITZ) [34]. This growth has positive significance for the interaction between recycled aggregate particles and the cementitious binder. As a result, the bonding effect—both between aggregates and between aggregates and the cementitious material—is enhanced [28]. Consequently, geopolymer-stabilized recycled aggregate achieves better cleavage strength than cement-stabilized recycled aggregate.

### 3.2. Durability Test

#### 3.2.1. Water Stability Test

**Figure 6:**
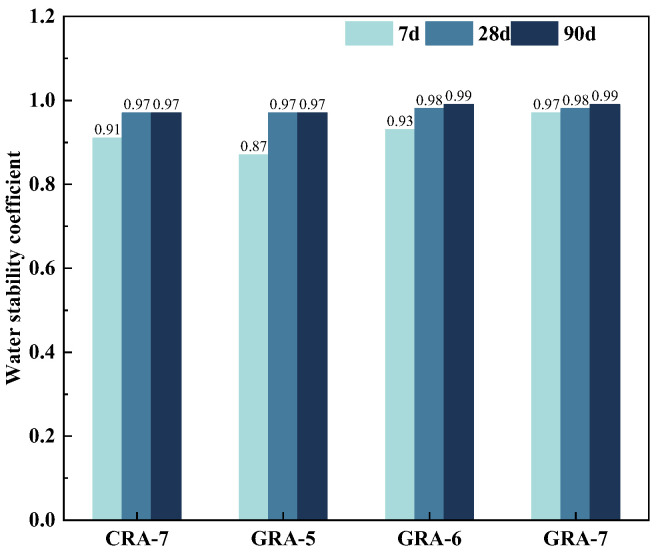
Water-stability test results of the samples.

Figure 6 and Table 4 show the test results of the water stability coefficient and the strength loss rate of the four groups of samples. The results showed that the water stability coefficient at 7 days decreased by 0.04 in the GRA-5 group compared to the CRA-7 group, while it increased by 0.02 and 0.06 in the GRA-6 and GRA-7 groups, respectively. However, the water stability coefficient of the 28-day and 90-day geopolymer test group was either the same or higher than that of the cement test group. The higher the water stability coefficient of the samples on the 7th and 28th days, the lower the strength loss rate, and the overall geopolymer test group showed excellent water stability. The test results showed that, compared with the CRA-7 group, the geopolymer significantly improved the recycled mixture’s water stability. This is because, in the early stage of curing, the geopolymer’s strength develops rapidly and the matrix structure forms, making it less affected by moisture. During cement hydration, the old mortar layer on the surface of the recycled aggregate participates little in the reaction. This results in weaker bonding between cement and recycled aggregate than between geopolymer and recycled aggregate [35]. Additionally, the old mortar layer contains more pores. In a water-rich environment, water is stored within these pores. This poses a potential threat to the interface between cement and recycled aggregate. Consequently, the water stability coefficient of cement-stabilized recycled aggregate is ultimately lower than that of geopolymer-stabilized recycled aggregate.

#### 3.2.2. Scouring Resistance

Table 5 presents the results of scouring resistance for the four sample groups. The overall results showed that the geopolymer group had better scouring resistance than the cement group. Scouring resistance improved with increasing geopolymer cementitious material content. The mass loss rate of the GRA-5, GRA-6, and GRA-7 groups decreased by 0.194%, 0.236%, and 0.315% compared with the CRA-7 group. The test results show that the geopolymer cementitious material can improve the overall compactness of the base material, form a dense matrix, reduce the water infiltration path, and thereby reduce mass loss under scouring. Secondly, the increase in cementitious material enhances adhesion between the cementitious material and the aggregate. The interface between the cementitious hydration products (such as C-S-H GEL and a three-dimensional network-like geopolymer structure) is more closely associated with the recycled aggregate [36]. The resistance to hydraulic peeling is improved. Finally, the increase in cementitious material content has a more pronounced effect on the scouring resistance of recycled aggregate stabilized with recycled polymer, because the polymer is more reactive and can quickly form a dense gel structure at low content. In contrast, the cement-stabilized recycled aggregate requires a higher dosage to compensate for the lack of coverage of surface defects by the cement hydration product [37]. Secondly, geopolymers form a three-dimensional aluminosilicate network structure through polycondensation reaction, which has better water resistance and chemical stability than cement-based materials. At the same time, geopolymers have a stronger ability to repair microcracks on the surface of recycled aggregates, which is conducive to reducing the weak points in interfacial transition zones (ITZ) [38].

### 3.3. Microscopic Testing

#### 3.3.1. MIP

**Figure 7:**
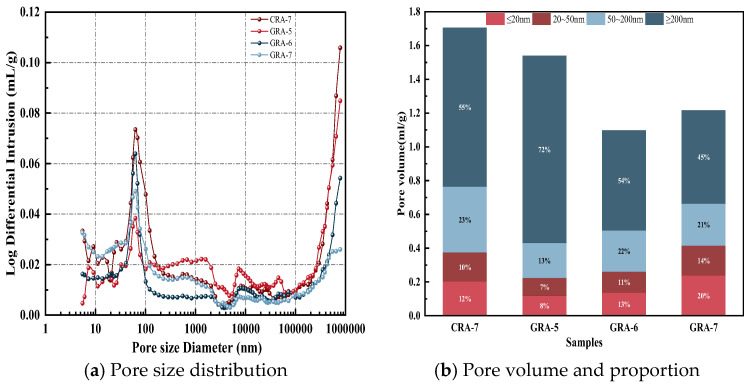
MIP experimental results of the samples.

Table 6 presents the total porosity test results for each sample group. Figure 7 shows the MIP test results of the four sets of samples. Based on the degree of harm of pores of different pore sizes and different distribution states to cementitious materials, pores are divided into the following four categories according to pore size: Class I: r ≤ 20 nm; Class II: 20 nm ≤ r ≤ 50 nm; Class III: 50 nm ≤ r ≤ 200 nm; Class IV: 200 nm ≤ r [39]. Among them, Class I and II pores cause only slight damage to materials. In contrast, Class III pores have the most significant impact on the drying and shrinkage properties of geopolymer composites and cement-based materials. In contrast, Class IV pores will negatively impact the mechanical properties of materials It can be seen from Figure 7a that the pore structure of the geopolymer test group transforms into small pores with the increase of the dosage, and the large pores in the GRA-7 group are reduced compared with the CRA-7 group under the exact dosage of 7%, and the pore structure is optimized. As shown in Figure 7b, with increasing geopolymer dosage, the proportion of Class I pores in the GRA-7 group increased by 5% and 12% compared with the GRA-5 and GRA-6 groups, respectively. The proportion of Class IV pores decreased by 18% and 27%, respectively. Under the exact dosage of 7%, the proportion of Class I pores in the GRA-7 group increased by 8% compared with that of the CRA-7 group, and the proportion of Class IV pores decreased by 10%. Replacing cement with geopolymers, or increasing their dosage, can effectively reduce the proportion of Class IV pores in the recycled mixture. This has a specific positive effect on the development of the mechanical properties of geopolymer-stabilized recycled aggregates. The reason may be that the reaction products in geopolymers consist mainly of gel phases, such as C-A-S-H and N-A-S-H. Unlike the hydration of ordinary silicate cements, which forms amorphous calcium silicate hydrate and crystalline phases (such as calcium hydroxide and ettringite), geopolymers contain no crystalline phases. Consequently, they lack nanoscale or micron-scale constraint effects [40]. This is particularly true during the early hydration stage, when crystalline products form less readily and amorphous gels predominate.

#### 3.3.2. SEM

**Figure 8:**
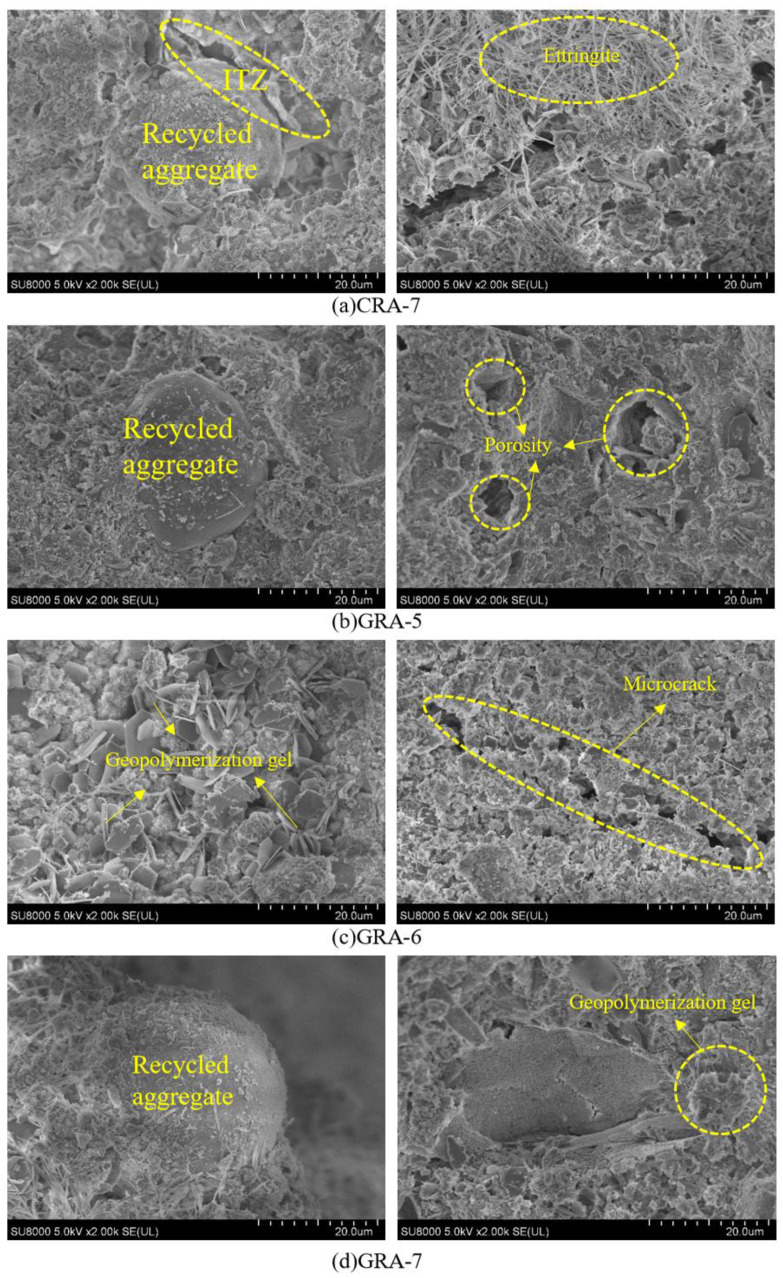
SEM images of internal aggregates and reaction products.

**Figure 9:**
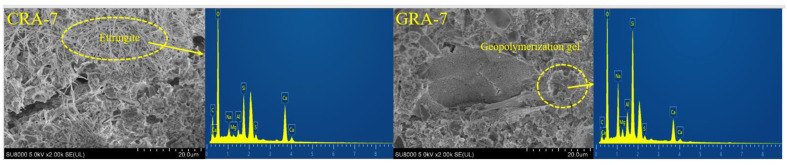
EDS results for CRA-7 and GRA-7 products.

Figure 8 shows SEM images of geopolymer-stabilized recycled aggregates and cement-stabilized recycled aggregates. Figure 9 shows the EDS analysis results for CRA-7 and GRA-7. Figure 8a shows a weak area between the recycled aggregate and the hydration product, with many microscopic pores and cracks and a relatively dispersed internal pore distribution [41], which has a negative impact on the subsequent development of mechanical properties. From Figure 8b–d, it can be seen that with increasing geopolymer content, the geopolymer cement around the recycled aggregate particles gradually increases. In the samples with 5% geopolymer content, the gel-phase pores around the recycled aggregate particles are more numerous and looser. In comparison, in the mixture samples with 7% geopolymer content, not only are a large number of flake gel products observed around the recycled aggregate [21], but also corresponding gel products are generated on the surface of the recycled aggregate particles, filling the pore structure [42]. This is the same as the MIP result. The surface of recycled aggregates contains aluminosilicate cement mortar, which dissolves in environments with high concentrations of OH^−^ ions, and partly participates in the hydration reaction, and the generated products are more closely linked to the recycled aggregate [43,44], which improves the performance of the sample, as reflected in its mechanical properties. The microstructure around recycled aggregate particles is looser and more porous than that of geopolymer-stabilized materials, further explaining why its mechanical properties are weaker than those of geopolymer-stabilized aggregate at the microscopic level.

#### 3.3.3. XRD

**Figure 10:**
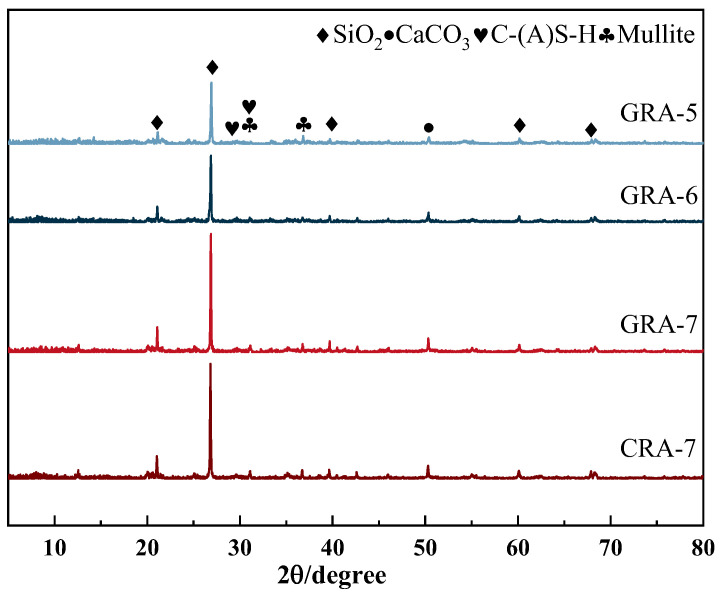
XRD test map of the samples.

Figure 10 shows the XRD profiles for the 4 sample groups. The figure shows the presence of SiO_2_, CaCO_3_, C-(A)-S-H, and mullite in each sample group [33]. Prominent diffraction peaks of SiO_2_ are observed between 20° and 30°, 60° and 70°, diffraction peaks of CaCO_3_ are observed at about 50°, respectively, and diffraction peaks of mullite can be observed between 30° and 40°. The diffraction peaks of the C-(A)-S-H gel appear between 25° and 35° degrees [45,46,47]. From the XRD spectrum, it is clear that the diffraction peaks of SiO_2_ near 27° and CaCO_3_ near 50° follow the same trend as the mechanical properties. The increase in geopolymer cementitious material leads to the formation of excessive C-(A)-S-H and CaCO_3_ [48], and the connectivity between hydration products and recycled aggregates increases, reflected in the sample’s mechanical strength. Under the exact dosage, the geopolymer experimental group generated more hydration products than the cement-based experimental group, which may be due to the erosion of the old mortar in the alkaline environment, and may partly participate in the reaction to participate in the hydration reaction [33], which increases the diffraction peak of some hydration products.

#### 3.3.4. TG-DTG

**Figure 11:**
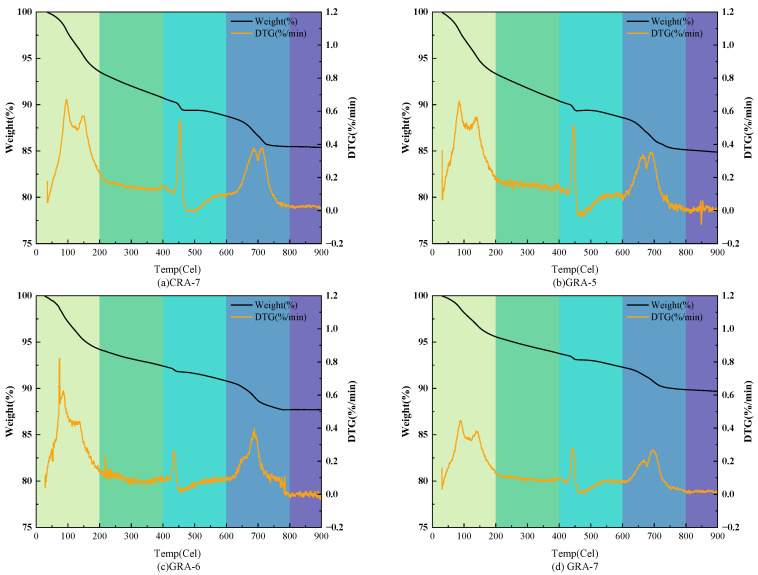
TG-DTG test results of the samples.

Figure 11 shows the TG-DTG test curve of the sample after 28 days of curing, and the test results indicate that the trend of the curve change is the same across the different test groups. With the increase of temperature, the weight loss rates of CRA-7, GRA-5, GRA-6, and GRA-7 experimental groups between 0~200 °C were 6.45%, 6.67%, 5.79% and 4.43%, respectively, which was due to the mass loss caused by the dehydration of C-(A)-S-H gel [49]. The weight loss rates between 400~600 °C were 11.76%, 11.41%, 9.21% and 7.71%, respectively, resulting from the decomposition of Ca (OH)_2_ [50]. The weight loss rates between 400~600 °C were 14.60%, 15.11%, 12.27% and 10.31%, respectively, which were caused by the decomposition of CaCO_3_ [51], and the mass loss rate of GRA-7 was 4.29% lower than that of CRA-7 under the same amount of cementitious material. Due to the lower dosage in the GRA-5 group, the amount of hydration products generated was lower than in the CRA-7 group. The GRA-6 and GRA-7 groups showed better stability due to higher dosage and greater hydration product generation, as observed in the XRD map, consistent with the compressive strength results.

## 4. Discussion

This study investigates the application of geopolymer as a cementitious binder replacement in crushed stone bases using a multi-component composite system. Results from mechanical and water resistance tests, combined with microscopic analysis, reveal why geopolymer binders outperform cementitious binders. Results indicate that under identical admixture ratios, the geopolymer binder generates more reaction products, improves specimen pore structure, reduces harmful pore volume, and narrows the interfacial transition zone (ITZ) width. However, water stability testing in this study remains limited. Current research on water stability in crushed stone bases lacks unified standards, and testing procedures lack standardized guidance.

In practical engineering applications, variations in material properties across regions may lead to differences in binder performance, increasing the cost of interregional material transportation. The distance between production sites and processing facilities also adds to material processing expenses. Production process limitations introduce impurities into the material, while immature sorting technology and high sorting costs restrict its large-scale application in actual projects. Future economic development will drive improvements in transportation and sorting technologies, potentially reducing costs and further enhancing the utilization value of solid waste materials.

## 5. Conclusions

In this paper, the unconfined compressive strength (UCS), splitting compressive strength (ITS), water stability, and erosion resistance of coal gangue powder (CGP), fly ash (FA) and granulated blast furnace slag powder (GGBS) ternary geopolymers were systematically studied, combined with mercury intrusion porosity (MIP), scanning electron microscopy (SEM) and X-ray diffraction (XRD) and thermogravimetry-derivative thermogravimetry (TG-DTG) analyzed the microscopic reaction mechanism of geopolymers. The following conclusions were drawn from the test results:

(1) The content of the geopolymer binder increased from 5% to 7%, and the UCS and ITS of the experimental group showed an increasing trend. In comparison, the UCS and ITS of the geopolymer test group increased by 7.58% and 9.52%, respectively, compared with the cement test group at the exact 7% binder dosage, indicating that the application of geopolymer in the crushed stone base layer has excellent potential.
(2) The water stability and erosion resistance of the geopolymer test group followed the same trend as UCS and ITS. In contrast, the strength loss in water stability and erosion resistance in the GRA-7 group was 1.000% and 0.315%, respectively, lower than those of the cement-based system, and the geopolymer system showed better stability.
(3) Microscopic test results indicate that under identical conditions of 7% binder dosage, the geopolymer test group exhibited an 8% increase in the proportion of harmless pores and a 10% decrease in the proportion of harmful pores compared to the cement control group. Concurrently, SEM and XRD analysis revealed more hydration products in the geopolymer test group and significant improvements in the interfacial transition zone, demonstrating superior stability. Compared to the cement control group, the geopolymer test group with the exact admixture dosage exhibited a 4.29% reduction in mass loss as temperature increased.

## Figures and Tables

**Table 1:** Chemical composition of cementitious materials.

Composition/wt%	SiO_2_	Al_2_O_3_	CaO	Na_2_O	MgO	Fe_2_O_3_	K_2_O	SO_3_	LOI
CGP	50.78	46.53	0.2	0.06	0.18	0.58	0.14	0.03	0.12
FA	51.53	20.45	7.15	1.63	1.94	5.81	1.87	1.76	5.94
GBFS	30.12	9.87	43.65	1.29	6.03	1.25	0.76	2.94	0.99
OPC	25.78	7.06	50.82	1.23	2.02	1.23	0.82	4.14	2.87

**Table 2:** Initial parameters of water glass solution.

Component	SiO_2_	Na_2_O	H_2_O	Initial Modulus
wt/%	23.49	9.41	67.10	2.58

**Table 3:** Test specimen mix ratio (kg/m^3^).

Trial Number	FA	CGP	GGBS	OPC	Recycled Aggregat	Water	Activator
CRA-7	-	-	-	155.833	2226.211	222.985	-
GRA-5	46.485	11.631	58.154	-	2326.214	-	264.799
GRA-6	55.611	13.902	69.513	-	2317.092	-	274.072
GRA-7	64.786	16.185	80.982	-	2313.749	-	289.620

**Table 4:** Strength loss rate.

Trial Number	Cementitious Material Content (%)	Loss of Strength (%)		
		7 d	28 d	90 d
CRA-7	7.0	9	3	3
GRA-5	5.0	13	3	2
GRA-6	6.0	7	2	1
GRA-7	7.0	3	2	1

**Table 5:** Results of anti-erosion experiments.

Trial Number	Specimen Mass (g)	Scouring Mass (g)	Washout Mass Loss (%)
CRA-7	6883.0	37.5	0.545
GRA-5	7164.8	25.1	0.351
GRA-6	7235.1	22.4	0.309
GRA-7	7309.0	16.8	0.230

**Table 6:** Total porosity of each sample group.

Trial Number	CRA-7	GRA-5	GRA-6	GRA-7
**Porosity**	20.6764	19.2239	13.4957	15.8899

## Data Availability

The original contributions presented in this study are included in the article. Further inquiries can be directed to the corresponding author.
